# Association between diabetes mellitus and anemia among Korean adults according to sex: a cross-sectional analysis of data from the Korea National Health and Nutrition Examination Survey (2010–2016)

**DOI:** 10.1186/s12902-021-00873-9

**Published:** 2021-10-21

**Authors:** Mihye Kim, Sook-Hyun Lee, Kyoung Sun Park, Eun-Jung Kim, Sujung Yeo, In-Hyuk Ha

**Affiliations:** 1 Jaseng Hospital of Korean Medicine, Seoul, Republic of Korea; 2 Jaseng Spine and Joint Research Institute, Jaseng Medical Foundation, 3F JS Tower, 538 Gangnam-daero, Gangnam-gu, Seoul, 06110 Republic of Korea; 3 Department of Acupuncture & Moxibustion, Dongguk University Bundang Oriental Hospital, Bundang, Republic of Korea; 4 Department of Meridian and Acupoint, College of Oriental Medicine, Sang Ji University, Wonju, Republic of Korea

**Keywords:** Diabetes mellitus, Anemia, Cross-sectional study, Korea National Health and Nutrition Examination Survey

## Abstract

**Background:**

There are many conflicting opinions regarding the association between anemia and diabetes mellitus (DM), and the mechanism by which DM influences anemia remains uncertain. Therefore, we aimed to investigate the association between anemia and DM in Korean adults and to analyze the risk factors for anemia among these patients according to sex.

**Methods:**

This retrospective cross-sectional survey was conducted using data from the Korea National Health and Nutrition Examination Survey V, VI, and VII between January 2010 and December 2016. In total, 25,597 Korean adults aged ≥19 years (10,117 men, 15,480 women) were included. Patients with a fasting blood sugar level of ≥126 mg/dL or who have been diagnosed with DM were classified as the DM group. Anemia was defined as hemoglobin levels of < 13 g/dL in men and < 12 g/dL in women. Logistic regression analysis was used to adjust for demographic characteristics and lifestyle-, disease-, and health-related factors.

**Results:**

Approximately 11.3% of patients had DM. The prevalence of anemia was significantly higher in the DM group than in the non-DM group. After adjusting for confounding factors, the odds of the prevalence of anemia in men were higher in the DM group than in the non-DM group (odds ratio [OR] 1.87, 95% confidence interval [CI] 1.42–2.50, *p* < 0.0001). When investigated according to the serum creatinine level, the association was significantly stronger among women (OR 42.63, 95% CI 17.25–105.36, *p* < 0.0001) than among men (OR 6.30, 95% CI 3.08–12.90, *p* < 0.0001).

**Conclusions:**

We found a strong association between DM and anemia that was more prominent among men than among women. We also determined that the serum creatinine level had a greater influence on DM and anemia in women than in men.

**Supplementary Information:**

The online version contains supplementary material available at 10.1186/s12902-021-00873-9.

## Background

Diabetes mellitus (DM) is a chronic disease that causes hyperglycemia because of impaired insulin secretion by pancreatic beta cells and peripheral insulin resistance, which may lead to serious complications [1]. As of 2019, the number of patients with DM worldwide was approximately 422 million, with an estimated prevalence of 8.5% [2]. The incidence of DM is increasing most rapidly among middle-to-low income countries [3], and it is predicted that one in 10 adults aged ≥20 years will have DM by 2045 [4]. Such a marked increase in the prevalence of DM could pose a significant economic burden on a country’s healthcare system. According to data from the Health Insurance Review and Assessment Service [5], the total cost of care for patients diagnosed with DM in the US was 573.7 billion won in 2019, an increase of 8.3% from that in the previous year [6].

Approximately 50–80% of patients with cardiovascular disease die, and DM may increase the risk of cardiovascular disease by increasing blood cholesterol and triglyceride levels. Accordingly, cardiovascular disease is considered a major cause of premature death, and its incidence among patients with DM reaches 20% after approximately 7 years [7]. Moreover, DM is often accompanied by acute or chronic complications, and because chronic complications are directly associated with patient death, prevention of such complications is of utmost importance.

Anemia, which is a major global public health issue, is a disease caused by the reduced ability to transport oxygen to tissues due to the lack of hemoglobin (Hb) [8]. Anemia is a common disease with a prevalence of nearly one-third of the world’s population, including that in Korea [9, 10]. It is more common among women than among men and in the older population than in the younger population [11]. The causes of anemia are complex and multifactorial; as it is a potential risk factor for cardiovascular disease, it may originate from complications related to various chronic diseases. However, there are many conflicting opinions about its association with DM.

Numerous studies on the association between DM and anemia have been published. Studies have demonstrated that anemia occurs in 14–45% of patients with DM [12, 13]. A study from the United States that analyzed adults aged ≥20 years reported that there is no association between DM and the onset of anemia among men or women [14]. This review of previous studies indicates that there are conflicting views on the association between anemia and DM, and the mechanism by which DM influences anemia remains uncertain.

Accordingly, we aimed to investigate the association between DM and anemia among Korean adults using nationally representative data from the Korea National Health and Nutrition Examination Survey (KNHANES) V, VI, and VII to analyze risk factors that could cause anemia in patients with DM. In particular, we sought to analyze sex-based differences in specific disease factors that could affect DM and anemia among Korean adults.

## Methods

### Study design and population

The present study used data from the KNHANES V, VI, and VII. The KNHANES is a cross-sectional survey conducted every 3 years to assess the health and nutritional status of the general Korean population. The survey includes a health screening, health questionnaire survey, and nutritional survey conducted with a nationally representative sample extracted by stratified, clustered, and systematic sampling methods [15].

**Fig. 1:**
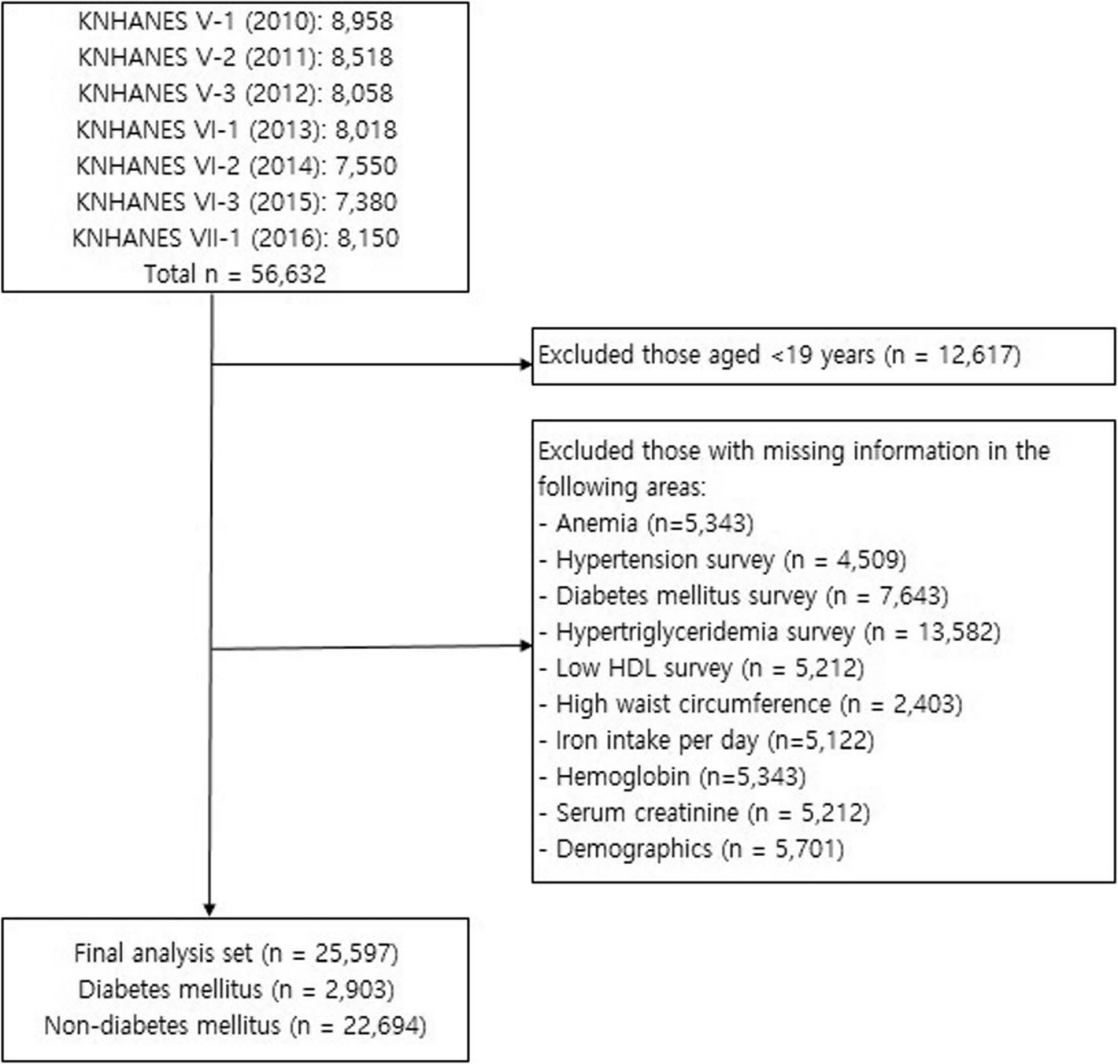
Inclusion and exclusion criteria for selecting participants from the 2010–2016 KNHANES V–VII HDL: high-density lipoprotein; KNHANES, Korea National Health and Nutrition Examination Survey

The study population in the present study comprised adults aged ≥19 years who took part in the health screening and health questionnaire survey among a sample population of 56,632 individuals who participated in the KNHANES, which was conducted between 2010 and 2016. The study population was limited by applying the following exclusion criteria: (a) age < 19 years (*n* = 12,617) and (b) missing values for anemia, hypertension, DM, hypertriglyceridemia, iron intake, Hb level, or serum creatinine level; a low high-density lipoprotein (HDL) cholesterol level; and a high waist circumference. After excluding 31,035 participants from a total of 56,632 individuals, the final data set consisted of 25,597 (45.2%) participants (Fig. 1).

### Outcomes and other variables

#### Measurement of DM

Based on the Korean Diabetes Association’s clinical practice guidelines (2016) [16], individuals with a fasting blood sugar (FBS) level of ≥126 mg/dL or who responded as having been diagnosed with DM were classified as the DM group, and those with an FBS level of < 126 mg/dL or who had not been diagnosed with DM were classified as the non-DM group.

#### Measurement of Hb levels

Hb level was used as the indicator of anemia. Based on the criteria put forth by the World Health Organization [17], anemia was defined as Hb levels of < 13 g/dL in men and < 12 g/dL in women.

#### Other covariates

We assessed the following covariates: demographic (including sex), lifestyle, disease, and health-related factors. Age was treated as a continuous variable, whereas sex was divided into men and women. Average monthly household income was divided into quartiles: low, lower middle, higher middle, and high. Education level was divided into four categories: below primary school, middle school, high school, and college or higher. Body mass index (BMI) was calculated as weight divided by height squared; underweight, normal weight, and overweight or obesity were defined as BMI of < 18.5 kg/m^2^, 18.5–24.9 kg/m^2^, and ≥ 25.0 kg/m^2^, respectively. Smoking status was divided into non−/ex-smoker and current smoker, and alcohol consumption was categorized as yes and no. For daily iron intake, data from the food-intake portion of the KNHANES were used, and the following criteria were applied: 10 mg for men aged 19–64 years, 9 mg for men aged ≥65 years, 14 mg for women aged 19–49 years, 8 mg for women aged 50–74 years, and 7 mg for women aged ≥75 years [18]. All blood samples were collected from the participants under fasting and analyzed within 24 h. Serum creatinine level and plasma glucose level were measured using hexokinase UV with a Hitachi 7600 Automatic Analyzer (Hitachi, Tokyo, Japan) [19]. The HbA1c level was analyzed using Tosoh G8 high-performance liquid chromatography (Tosoh, Tokyo, Japan). Hypertension was defined as a blood pressure of > 140/90 mmHg. Hypertriglyceridemia [20] was defined as a triglyceride level of ≥200 mg/dL, which indicates classification into the high or very high group according to the National Cholesterol Education Program Adult Treatment Panel III (NCEP ATP III). Low HDL cholesterol levels were defined as < 40 mg/dL for men and < 50 mg/dL for women.

### Statistical analysis

Because the KNHANES contains data based on a complex sample design, complex-sample statistical analysis was used with consideration of weight, stratification variables, and cluster variables. Differences in the characteristics of the study population depending on DM and anemia were analyzed using the Rao-Scott chi-square test or t-test. Data of categorical variables are expressed as frequency and percentage (%), whereas data of continuous variables are expressed as mean ± standard error.

To analyze the associations with anemia depending on DM, logistic regression analysis was performed to derive the odds ratio (OR) and confidence interval (CI). Logistic regression was used to assess the impact of DM, sex, and the interaction between DM and sex on developing anemia and to calculate OR. Additionally, subgroup analyses were performed on factors that may influence anemia in patients with DM by sex (men versus women). SAS, version 9.4 (SAS Institute Inc., Cary, NC), was used for all statistical analyses. The significance level was set to *p*-values of < 0.05 for all tests.

## Results

Table 1 shows the characteristics of the study population depending on DM. There were 2903 participants (11.3%) in the DM group and 22,694 participants (88.7%) in the non-DM group and more women (*n* = 1486) than men (*n* = 1417; 48.8%). Mean patient age in the DM group was 60.09 ± 0.31 years, which was higher than that in the non-DM group. With regard to education level, “below primary school” was the most common response (*n* = 1299; 44.8%) in the DM group, whereas “high school” was the most common response (*n* = 7668; 33.8%) in the non-DM group (*p* < 0.0001). Mean BMI (kg/m^2^) was higher in the DM group (25.3 ± 0.1) than in the non-DM group (23.6 ± 0.03) (*p* < 0.0001); there were 1490 (51.3%) participants with a normal weight in the DM group and 14,766 (65.1%) in the non-DM group, showing higher percentages than both the underweight and overweight and obesity subgroups (*p* < 0.0001). There were more non−/ex-smokers in the DM group (*n* = 18,983; 83.7%) than in the non-DM group (*n* = 2352; 81%) (*p* = 0.0076). With regard to alcohol consumption, the number of participants who responded “yes” was 2284 (78.7%) in the DM group and 19,781 (87.2%) in the non-DM group, which was much higher than the number of participants who responded “no” (*p* < 0.0001).

**Table 1:** Characteristics of the study population with and without diabetes mellitus

Variables	Non-DM (*n* = 22,694)	DM (*n* = 2903)	*p*-value
Age (years) ^a^	45.28 ± 0.13	60.09 ± 0.31	<.0001
Sex ^b^			
Men	8700 (38.3)	1417 (48.8)	<.0001
Women	13994 (61.7)	1486 (51.2)	
Household income ^b^			
Low	4082 (18)	1013 (34.9)	<.0001
Lower middle	5780 (25.5)	796 (27.4)	
Higher middle	6380 (28.1)	588 (20.3)	
High	6452 (28.4)	506 (17.4)	
Educational level ^b^			
≤ Elementary school	5168 (22.8)	1299 (44.8)	<.0001
Middle school	2320 (10.2)	449 (15.5)	
High school	7668 (33.8)	750 (25.8)	
≥ College	7538 (33.2)	405 (14)	
BMI (kg/m2) ^a, b^	23.6 ± 0.03	25.27 ± 0.09	<.0001
< 18.5 (underweight)	1005 (4.4)	36 (1.2)	<.0001
< 25 (normalweight)	14766 (65.1)	1490 (51.3)	
≥ 25 (overweight or obese)	6923 (30.5)	1377 (47.4)	
Smoking status ^b^			
Non−/Ex-smoker	18,983 (83.7)	2352 (81)	0.0076
Current smoker	3711 (16.4)	551 (19)	
Alcohol consumption ^b^			
No	2913 (12.8)	619 (21.3)	<.0001
Yes	19,781 (87.2)	2284 (78.7)	
Non-Anemia	20,764 (91.5)	2575 (88.7)	<.0001
Anemia	1930 (8.5)	328 (11.3)	<.0001
Iron intake per day (mg/day) ^a^	16.68 ± 0.16	16.66 ± 0.38	0.9651
Hemoglobin (g/dL) ^a^	14.12 ± 0.01	14.14 ± 0.04	0.6599
Serum creatinine (mg/dL) ^a^	0.83 ± 0.00	0.9 ± 0.01	<.0001
Waist circumference (cm) ^a^	80.56 ± 0.08	87.53 ± 0.23	<.0001
High waist circumference ^b^	5481 (24.2)	1360 (46.9)	<.0001
HTN ^b^	6487 (28.6)	1822 (62.8)	<.0001
Fasting glucose level (mg/dL) ^a^	92.96 ± 0.08	142.84 ± 1.01	<.0001
HbA1c (%) ^a’^	5.53 ± 0.00	7.36 ± 0.04	<.0001
Hypertriglyceridemia ^b^	2924 (12.9)	777 (26.8)	<.0001
Low HDL cholesterol ^b^	8151 (35.9)	1562 (53.8)	<.0001

^a^ Continuous variables are presented means ± standard error and compared using t-test

^b^ Categorical variables are presented as frequencies and percentages and compared using the Rao-Scott Chi-Square test

***DM*** diabetes mellitus ***BMI*** body mass index; ***HTN*** hypertension; ***HbA1c*** glycated hemoglobin; ***HDL*** high-density lipoprotein

As for clinical factors that influence DM, the DM group showed a higher percentage and mean value for waist circumference, a high waist circumference, FBS level (mg/dL), HbA1c level, hypertension, hypertriglyceridemia, and a low HDL cholesterol level than the non-DM group (*p* < 0.0001). However, there were no significant differences in daily iron intake or Hb level.

Table 2 shows the characteristics of the study population depending on diabetes and anemia. There were 2258 participants (8.9%) in the anemia group and 23,339 (91.1%) in the non-anemia group, and there were more women (*n* = 1796, 79.5%) than men in the anemia group (*n* = 462, 20.5%) (*p* < 0.0001). Mean patient age in the anemia group with DM was 65.93 ± 0.86 years, which was higher than that in the non-anemia group in DM and all groups in non-DM (*p* < 0.0001). Regarding household income, the prevalence of anemia tended to be higher among the lower income quartiles in the DM group, whereas it tended to increase with an increasing income level in the non-DM group.

**Table 2:** Characteristics according to with or without of diabetes and anemia

Variables	Non-DM			DM		
	Non-anemia	Anemia	*p*-value	Non-anemia	Anemia	*p*-value
	(*n* = 20,764)	(*n* = 1930)		(*n* = 2575)	(*n* = 328)	
Age (years) ^a^	45.08 ± 0.18	47.57 ± 0.46	<.0001	59.36 ± 0.33	65.93 ± 0.86	<.0001
Sex ^b^						
Men	8376 (40.3)	324 (16.8)	<.0001	1279 (49.7)	138 (42.1)	<.0001
Women	12388 (59.7)	1606 (83.2)		1296 (50.3)	190 (57.9)	
Household income ^b^						
Low	3661 (17.6)	421 (21.8)	<.0001	876 (34.0)	137 (41.8)	0.0095
Lower middle	5271 (25.4)	509 (26.4)		705 (27.4)	91 (27.7)	
Higher middle	5854 (28.2)	526 (27.3)		537 (20.9)	51 (15.6)	
High	5978 (28.8)	474 (24.6)		457 (17.8)	49 (14.9)	
Educational level ^b^						
≤ Elementary school	4671 (22.5)	497 (25.8)	<.0001	1118 (43.4)	181 (55.2)	<.0001
Middle school	2160 (10.4)	160 (8.3)		411 (16.0)	38 (11.6)	
High school	6990 (33.7)	678 (35.1)		672 (26.1)	78 (23.8)	
≥ College	6943 (33.4)	595 (30.8)		374 (14.5)	31 (9.5)	
BMI (kg/m^2^) ^a, b^	23.67 ± 0.03	22.68 ± 0.09	<.0001	25.37 ± 0.09	24.46 ± 0.42	<.0001
< 18.5 (underweight)	864 (4.2)	141 (7.3)	<.0001	22 (0.9)	14 (4.3)	<.0001
< 25 (normoweight)	13,409 (64.6)	1357 (70.3)		1294 (50.3)	196 (59.8)	
≥ 25 (overweight or obese)	6491 (31.3)	432 (22.4)		1259 (48.9)	118 (36)	
Smokers ^b^						
Non−/Ex-smoker	17151 (82.6)	1832 (94.9)	<.0001	2066 (80.2)	286 (87.2)	0.0001
Current smoker	3613 (17.4)	98 (5.1)		509 (19.8)	42 (12.8)	
Alcohol consumption ^b^						
No	2598 (12.5)	315 (16.3)	<.0001	533 (20.7)	86 (26.2)	0.0068
Yes	18166 (87.5)	1615 (83.7)		2042 (79.3)	242 (73.8)	
Iron intake per day (mg/day) ^a^	16.19 ± 0.17	14.06 ± 0.26	<.0001	16.31 ± 0.43	12.68 ± 0.52	<.0001
Hemoglobin (g/dL) ^a^	14.37 ± 0.01	11.02 ± 0.03	<.0001	14.47 ± 0.03	11.31 ± 0.07	<.0001
Serum creatinine (mg/dL) ^a^	0.83 ± 0.002	0.8 ± 0.02	<.0001	0.87 ± 0.005	1.23 ± 0.13	<.0001
Waist circumference (cm) ^a^	80.85 ± 0.1	76.97 ± 0.26	<.0001	87.85 ± 0.24	84.82 ± 0.88	<.0001
High waist circumference ^b^	5151 (24.8)	330 (17.1)	<.0001	1242 (48.2)	118 (36.0)	0.0003
Fasting glucose level (mg/dL) ^a^	93.09 ± 0.1	91.3 ± 0.24	<.0001	144.13 ± 1.05	131.83 ± 3.09	<.0001
HTN ^b^	5967 (28.7)	520 (26.9)	0.1012	1584 (61.5)	238 (72.6)	0.0008
HbA1c (%) ^a^	5.52 ± 0.005	5.62 ± 0.01	<.0001	7.37 ± 0.04	7.28 ± 0.1	<.0001
Hypertriglyceridemia ^b^	2816 (13.6)	108 (5.6)	<.0001	719 (27.9)	58 (17.7)	<.0001
Low HDL cholesterol ^b^	7345 (35.4)	806 (41.8)	<.0001	1366 (53.1)	196 (59.8)	0.017

^a^ Continuous variables are presented means ± standard error and compared using t-test

^b^ Categorical variables are presented as frequencies and percentages and compared using the Rao-Scott Chi-Square test

***BMI*** body mass index; ***HTN*** hypertension; ***DM*** diabetes mellitus; ***HbA1c*** glycated hemoglobin; ***HDL*** high-density lipoprotein

With regard to education level in the non-DM group, “high school” was the most common response in both the anemia (*n* = 678, 35.1%) and non-anemia (*n* = 6990, 33.7%) groups (*p* < 0.0001). In contrast, the most common education level in the DM group was “≤Elementary school” in both the anemia (*n* = 181, 55.2%) and non-anemia (*n* = 1118, 43.4%) groups (*p* < 0.0001). Mean BMI (kg/m^2^) was higher in the non-anemia group than in the anemia group in both the non-DM and DM groups (*p* < 0.0001). In the anemia group, the proportion of current smokers in the DM group (12.8%) was relatively higher than that in the non-DM group (5.1%) (*p* < 0.0001). Regarding clinical factors that influence anemia, there were significant differences in the daily iron intake (mg/day), Hb level (g/dL), serum creatinine level (mg/dL), waist circumference, high waist circumference, and fasting glucose level (mg/dL) between the two groups.

Table 3 shows the characteristics of the study population depending on anemia by sex in DM. Among men, there were significant differences between the anemia and non-anemia groups except in alcohol consumption and low HDL cholesterol factors. Among women, there were significant differences between the groups in age, mean BMI, daily iron intake, Hb level, serum creatinine level, waist circumference, high waist circumference, fasting glucose level, and HbA1c level.

**Table 3:** Characteristics of the study population with and without anemia according to sex in diabetes mellitus

Variables	Men			Women		
	Non-anemia	Anemia	*p*-value	Non-anemia	Anemia	*p*-value
	(*n* = 1279)	(*n* = 138)		(*n* = 1296)	(*n* = 190)	
Age (years) ^a^	57.23 ± 0.42	68.09 ± 0.91	<.0001	61.91 ± 0.45	64.58 ± 1.28	<.0001
Household income ^b^						
Low	371 (29)	56 (40.6)	0.0031	505 (39)	81 (42.6)	0.4453
Lower middle	350 (27.4)	39 (28.3)		355 (27.4)	52 (27.4)	
Higher middle	284 (22.2)	24 (17.4)		253 (19.5)	27 (14.2)	
High	274 (21.4)	19 (13.8)		183 (14.1)	30 (15.8)	
Educational level ^b^						
≤ Elementary school	357 (27.9)	47 (34.1)	0.0075	761 (58.7)	134 (70.5)	0.2327
Middle school	235 (18.4)	13 (9.4)		176 (13.6)	25 (13.2)	
High school	407 (31.8)	55 (39.9)		265 (20.5)	23 (12.1)	
≥ College	280 (21.9)	23 (16.7)		94 (7.3)	8 (4.2)	
BMI (kg/m^2^) ^a, b^	25.1 ± 0.12	23.2 ± 0.31	<.0001	25.69 ± 0.13	25.24 ± 0.64	<.0001
< 18.5 (underweight)	8 (0.6)	8 (5.8)	<.0001	14 (1.1)	6 (3.2)	0.105
< 25 (normoweight)	693 (54.2)	101 (73.2)		601 (46.4)	95 (50)	
≥ 25 (overweight or obese)	578 (45.2)	29 (21)		681 (52.6)	89 (46.8)	
Smokers ^b^						
Non−/Ex-smoker	825 (64.5)	106 (76.8)	0.0008	1241 (95.8)	180 (94.7)	0.6657
Current smoker	454 (35.5)	32 (23.2)		55 (4.2)	10 (5.3)	
Alcohol consumption ^b^						
No	81 (6.3)	12 (8.7)	0.1235	452 (34.9)	74 (39)	0.4257
Yes	1198 (93.7)	126 (91.3)		844 (65.1)	116 (61.1)	
Iron intake per day (mg/day) ^a^	18.09 ± 0.68	14.05 ± 0.61	<.0001	14.18 ± 0.43	11.83 ± 0.78	<.0001
Hemoglobin (g/dL) ^a^	15.27 ± 0.04	11.79 ± 0.12	<.0001	13.51 ± 0.03	11.01 ± 0.09	<.0001
Serum creatinine (mg/dL) ^a^	0.98 ± 0.01	1.68 ± 0.32	<.0001	0.73 ± 0	0.95 ± 0.04	<.0001
Waist circumference (cm) ^a^	88.83 ± 0.32	84.75 ± 0.92	<.0001	86.68 ± 0.33	84.86 ± 1.32	<.0001
High waist circumference ^b^	540 (42.2)	34 (24.6)	0.0002	702 (54.2)	84 (44.2)	0.0068
HTN ^b^	743 (58.1)	98 (71)	<.0001	841 (64.9)	140 (73.7)	0.3057
Fasting glucose level (mg/dL) ^a^	147.17 ± 1.55	124.51 ± 3.24	<.0001	140.5 ± 1.48	136.41 ± 4.53	<.0001
HbA1c (%) ^a^	7.4 ± 0.06	7.08 ± 0.12	<.0001	7.33 ± 0.05	7.41 ± 0.13	<.0001
Hypertriglyceridemia ^b^	417 (32.6)	19 (13.8)	<.0001	302 (23.3)	39 (20.5)	0.6261
Low HDL cholesterol ^b^	519 (40.6)	61 (44.2)	0.4338	847 (65.4)	135 (71.1)	0.3557

^a^ Continuous variables are presented means ± standard error and compared using t-test

^b^ Categorical variables are presented as frequencies and percentages and compared using the Rao-Scott Chi-Square test

***BMI*** body mass index; ***HTN*** hypertension; ***DM*** diabetes mellitus; ***HbA1c*** glycated hemoglobin; HDL, high-density lipoprotein

Table 4 shows the association between DM and anemia analyzed using logistic regression analyses by sex; the results were significant in both the unadjusted model (Model 1) and adjusted models (Models 2 and 3) for men. In particular, the ORs for anemia in the DM group were higher than those in the non-DM group by 3.49-fold (95% CI 2.69–4.53, *p* < 0.0001), 2.18-fold (95% CI 1.67–2.85, *p* < 0.0001), and 1.87-fold (95% CI 1.42–2.50, *p* < 0.0001) in Models 1, 2, and 3, respectively. For women, the results were not significant in the unadjusted model (Model 1), although they were significant in the adjusted models (Models 2 and 3). In particular, the ORs for anemia were higher in the DM group than those in the non-DM group by 1.31-fold (95% CI 1.07–1.60, *p* = 0.0093) and 1.33-fold (95% CI 1.09–1.63, *p* = 0.0067) in Models 2 and 3, respectively. Compared to the group without a history of DM, OR for the incident DM was significantly higher in an unadjusted model (OR: 3.49; 95% CI: 2.69–4.53); the association observed remained significant in Model 2 (OR: 3.10; 95% CI: 2.38–4.04) and Model 3 (OR: 2.97; 95% CI: 2.27–3.89). Among men (the reference category for sex), compared to the group without a history of DM, OR for the incident DM was significantly higher in an unadjusted model (OR: 3.49; 95% CI: 2.69–4.53); the association observed remained significant in Model 2 (OR: 3.10; 95% CI: 2.38–4.04) and Model 3 (OR: 2.97; 95% CI: 2.27–3.89).

**Table 4:** Logistic regression analyses of the association between anemia and DM by sex

	Model 1			Model 2	Model 3				
	OR ^a^	95% CI	*p*-value	OR ^a^	95% CI	*p*-value	OR ^a^	95% CI	*p*-value
**Men**									
Non-DM	1			1			1		
DM	3.49	2.69–4.53	<.0001	2.18	1.67–2.85	<.0001	1.87	1.42–2.50	<.0001
**Women**									
Non-DM	1			1			1		
DM	1.21	0.99–1.48	0.0611	1.31	1.07–1.60	0.0093	1.33	1.09–1.63	0.0067

Model 1: unadjusted

Model 2: adjusted for age, sex, household income, educational level, body mass index, smoking status, and alcohol consumption

Model 3: adjusted for age, sex, household income, educational level, body mass index, smoking status, alcohol consumption, iron intake per day, serum creatinine level, high waist circumference, hypertension, hypertriglyceridemia, and low high-density lipoprotein cholesterol level

^a^ Odds ratios with adjustments using logistic regression models

***DM*** diabetes mellitus; ***OR*** odds ratio; ***CI*** confidence interval

Moreover, among those with no DM (the reference category for diabetes), men had a 5.52-fold higher OR for anemia (95% CI 4.73–6.45, *p* < 0.0001) in Model 1, 4.36-fold higher OR (95% CI 3.70–5.14, *p* < 0.0001) in Model 2, 5.66-fold higher OR (95% CI 4.61–6.95, *p* < 0.0001) in Model 3 than women.

Lastly, our study demonstrated a significant association in the interaction between sex and DM on the development of anemia in Model 1 (OR: 0.35; 95% CI: 0.25–0.49, *p* < 0.0001), Model 2 (OR: 0.37; 95% CI: 0.27–0.52, *p* < 0.0001), and Model 3 (OR: 0.39; 95% CI: 0.27–0.54, *p* < 0.0001) in Additional file 1. This can be interpreted as the odds for anemia for those with DM versus no DM being higher for men (the reference category for sex) than for women.

Table 5 shows the results of logistic regression analyses on regarding the influence of risk factors on the prevalence of anemia among patients with DM as the change in the odds ratio for a unit change in the continuous variables. The results were significant for daily iron intake, serum creatinine level, high waist circumference, and hypertriglyceridemia in Models 1, 2, and 3. In particular, the serum creatinine level showed ORs of 6.28 (95% CI 3.95–9.99, *p* < 0.0001), 14.28 (95% CI 8.15–25.01, *p* < 0.0001), and 13.40 (95% CI 7.64–23.51, *p* < 0.0001) in Models 1, 2, and 3, respectively, which indicates that the serum creatinine level is a highly influential risk factor for anemia in patients with DM.

**Table 5:** Odds ratios for risk factors of anemia in patients with diabetes by sex

	Model 1			Model 2			Model 3		
	OR ^a^	95% CI	*p*-value	OR ^a^	95% CI	*p*-value	OR ^a^	95% CI	*p*-value
**Total**									
Iron intake per day (mg/day)	0.96	0.95–0.98	<.0001	0.98	0.96–0.99	0.0029	0.98	0.97–1.00	0.0137
Serum creatinine (mg/dL)	6.28	3.95–9.99	<.0001	14.28	8.15–25.01	<.0001	13.40	7.64–23.51	<.0001
High waist circumference	0.59	0.44–0.79	0.0004	0.52	0.31–0.88	0.015	0.47	0.26–0.83	0.0097
HTN	1.69	1.25–2.29	0.0007	1.30	0.94–1.79	0.1133	1.09	0.78–1.53	0.6134
Hypertriglyceridemia	0.49	0.34–0.70	<.0001	0.69	0.42–0.90	0.0129	0.62	0.41–0.92	0.0179
Low HDL cholesterol	1.41	1.064–1.87	0.017	1.23	0.92–1.65	0.1592	1.27	0.93–1.73	0.1307
**Men**									
Iron intake per day (mg/day)	0.96	0.94–0.98	0.0005	0.98	0.96–1.00	0.02	0.98	0.96–1.00	0.0744
Serum creatinine (mg/dL)	10.31	5.40–19.70	<.0001	8.00	3.84–16.65	<.0001	6.30	3.08–12.90	<.0001
High waist circumference	0.42	0.26–0.67	0.0003	0.73	0.37–1.44	0.3597	0.65	0.31–1.35	0.2458
HTN	2.49	1.61–3.85	<.0001	2.14	1.31–3.49	0.0025	1.86	1.18–3.13	0.0191
Hypertriglyceridemia	0.24	0.13–0.43	<.0001	0.38	0.20–0.72	0.003	0.40	0.21–0.77	0.0065
Low HDL cholesterol	1.17	0.79–1.73	0.4322	1.32	0.85–2.06	0.2178	1.34	0.88–2.20	0.2462
**Women**									
Iron intake per day (mg/day)	0.98	0.95–1.00	0.042	0.98	0.96–1.00	0.0451	0.98	0.96–1.00	0.1147
Serum creatinine (mg/dL)	34.50	13.98–85.12	<.0001	37.12	15.35–89.76	<.0001	42.63	17.25–105.36	<.0001
High waist circumference	0.61	0.42–0.89	0.01	0.49	0.28–0.87	0.0149	0.38	0.21–0.71	0.0022
HTN	1.25	0.83–1.86	0.2835	1.06	0.70–1.60	0.7868	0.89	0.58–1.35	0.5721
Hypertriglyceridemia	0.89	0.56–1.40	0.6152	0.89	0.56–1.41	0.6096	0.86	0.52–1.42	0.5518
Low HDL cholesterol	1.22	0.81–1.85	0.349	1.20	0.81–1.77	0.3736	1.22	0.80–1.86	0.3684

Model 1: unadjusted

Model 2: adjusted for age, sex, household income, educational level, body mass index, smoking status, and alcohol consumption

Model 3: adjusted for age, sex, household income, educational level, body mass index, smoking status, alcohol consumption, iron intake per day, serum creatinine level, high waist circumference, hypertension, hypertriglyceridemia, and low HDL cholesterol level

^a^ Odds ratios with adjustments using logistic regression models

***HTN*** hypertension; ***HDL*** high-density lipoprotein; ***OR*** odds ratio; ***CI*** confidence interval

A high waist circumference showed significant ORs of 0.59 (95% CI 0.44–0.79, *p* = 0.0004), 0.52 (95% CI 0.31–0.88, *p* = 0.015), and 0.47 (95% CI 0.26–0.83, *p* = 0.0097) in Models 1, 2, and 3, respectively. Hypertriglyceridemia also showed significant ORs of 0.49 (95% CI 0.34–0.70, *p* < 0.0001), 0.69 (95% CI 0.42–0.90, *p* = 0.0129), and 0.62 (95% CI 0.41–0.92, *p* = 0.0179) in Models 1, 2, and 3, respectively.

When the influence of risk factors on the prevalence of anemia among patients with DM was examined by sex, the results showed slight differences between men and women. Among men, the serum creatinine level, hypertension, and hypertriglyceridemia were significant factors in both the unadjusted (Model 1) and adjusted (Models 2 and 3) models. The serum creatinine level showed significant ORs of 10.31 (95% CI 5.40–19.70, *p* < 0.0001), 8.00 (95% CI 3.84–16.65, *p* < 0.0001), and 6.30 (95% CI 3.08–12.90, *p* < 0.0001) in Models 1, 2, and 3, respectively. Hypertension also showed significant ORs of 2.49 (95% CI 1.61–3.85, *p* < 0.0001), 2.14 (95% CI 1.31–3.49, *p* = 0.0025), and 1.86 (95% CI 1.18–3.13, *p* = 0.0191) in Models 1, 2, and 3, respectively. Additionally, hypertriglyceridemia showed significant ORs of 0.24 (95% CI 0.13–0.43, *p* < 0.0001), 0.38 (95% CI 0.20–0.72, *p* = 0.003), and 0.40 (95% CI 0.21–0.77, *p* = 0.0065) in Models 1, 2, and 3, respectively.

Among women, the serum creatinine level and a high waist circumference showed significant ORs in Models 1, 2, and 3 (OR = 34.50, 95% CI 13.98–85.12, *p* < 0.0001 in Model 1; OR = 37.12, 95% CI 15.35–89.76, *p* < 0.0001 in Model 2; and OR = 42.63, 95% CI 17.25–105.36, *p* < 0.0001 in Model 3). A high waist circumference alone showed significant ORs of 0.61 (95% CI 0.42–0.89, *p* = 0.01), 0.49 (95% CI 0.28–0.87, *p* = 0.0149), and 0.38 (95% CI 0.21–0.71, *p* = 0.0022) in Models 1, 2, and 3, respectively.

## Discussion

The present study used nationally representative and highly reliable data from the KNHANES V, VI, and VII to investigate the association between DM and anemia among Korean adults aged ≥19 years. Our findings indicated a clear association between DM and anemia among Koreans. In full adjusted Model 3, the OR for anemia in the DM group increased by 2.97-fold as compared to the OR for the non-DM group. Accordingly, it was determined that the DM group had a more significant association with anemia than the non-DM group (*p* < 0.0001).

With regard to previous studies on DM and anemia, a study conducted among adults from the United States reported no association between DM and anemia, regardless of sex, which was in line with the conclusion drawn in a study from Spain that analyzed patients on nephrology dialysis also reported no association between DM and anemia [14, 21]. However, findings of the present study indicated an association between DM and anemia by sex, which is supported by findings from other studies on the association between iron-deficiency anemia and HbA1c level in DM^,^ and the association between anemia and kidney function in patients with DM.^,^ However, those studies used different diagnostic criteria for anemia and included participants of different ages and ethnic groups; thus, it is difficult to make direct comparisons between studies.

A previous Korean study [22] investigated the influence of DM on the onset of anemia among Korean adults, but the study found that after adjusting for other risk factors for anemia, DM was not an independent risk factor for anemia; this is contrary to the findings of the present study. Even when examining by sex, another Korean study [18] analyzed the OR of DM on the onset of anemia among Korean adults and found that DM increased the OR for anemia by 2.6-fold among men after adjusting for age, education level, monthly household income, smoking status, alcohol consumption, iron intake, and disease history, whereas DM did not influence the onset of anemia among women.

The present study also investigated the influence of risk factors on the prevalence of anemia among patients with DM by sex. Our study found that men had a 5.66-fold higher OR for anemia than women, and there was a significant association in interaction between sex and DM on the development of anemia (ORs of 0.39 in Model 3).

Recent studies have identified the associations of chronic kidney failure and elevated creatinine levels with metabolic syndrome, including DM [23]. The results showed that the serum creatinine level was a significant risk factor for anemia in both men and women. In particular, the serum creatinine level showed ORs of 6.30 (*p* < 0.0001) in men and 42.63 (*p* < 0.0001) in women after adjusting for demographic, lifestyle, disease, and health-related factors, which indicates that the serum creatinine level greatly influences the prevalence of anemia, especially among women with DM.

The association between serum creatinine level and anemia in patients with DM was a noteworthy finding in the present study. A previous study [24] found no significant association between serum creatinine level (mg/dL) and anemia among patients with DM, as well as no significant association with GFR (mL/min). In the present study, however, the serum creatinine level showed a strong association with anemia in women with DM. Such a difference could be attributed to the fact that the existing report was a case study that recruited patients with DM and had a sample size of only 142, whereas the present study analyzed data from 25,597 people. In addition, the existing study particularly recruited patients with type 2 DM, whereas the KNHANES included individuals with type 1 and type 2 DM. Additional studies are needed to investigate the mechanisms involved in DM, anemia, and kidney disease by sex.

The present study had some limitations because it used KNHANES data [25]. First, as the KNHANES was a cross-sectional survey study, the association between DM and anemia could be examined, although the causal relationship between DM and anemia must be interpreted with caution. Second, a major limitation in studying anemia is that the onset involves diverse and complex mechanisms; however, the KNHANES defined anemia simply based on the Hb level. Therefore, it was difficult to identify other risk factors for anemia such as vitamin B_12_ level. Moreover, chronic anemia and iron-deficiency anemia cannot be differentiated by the present study alone. Third, when defining the covariates hypertension and hyperlipidemia, this study defined only the numbers without considering whether participants were taking medication during the study period to lower their levels. Hypertension and hyperlipidemia are covariates rather than direct outcomes in this paper, and the two diseases are chronic diseases and belong to the related cardiovascular system. If each disease included people on medication, then it would be difficult to distinguish each group, which may have affected the significance of the results; therefore, people on medication were not considered. Another limitation of this study is that people whose diseases were being numerically controlled through drugs were not considered. Fourth, the number of patients with diabetic nephropathy among the included patients with DM could not be identified using the KNHANES data, making it difficult to accurately identify the association. Although the KNHANES provides nationally representative data, the survey itself did not focus on DM and anemia. As such, there is a lack of data on biological values such as those on the GFR and blood erythropoietin level, which could be used to determine kidney health [26].

While it is unclear whether correcting anemia is helpful for patients with DM, DM is known to cause various complications, such as diabetic nephropathy and cardiovascular disease, and increase hospitalization and mortality rates. Therefore, continued management and treatment of DM are important. Future studies should compare patients with kidney disease caused by DM and patients with DM who do not have kidney disease.

Our findings showed that, after adjusting for all other risk factors for anemia, DM was an independent risk factor for anemia among Korean men. Therefore, anemia should be monitored not only in women and malnourished patients but also in patients with DM. The management and treatment of anemia are important for preventing DM-related complications such as kidney disease and cardiovascular disease. Despite the limitations, the present study contributes to the existing literature in that it investigated the association between DM and anemia within the Korean population. This study is the first to investigate sex-based differences in factors that influence DM and anemia. Large-scale studies that overcome the limitations of the present study are needed in the future, and the findings of the present study may serve as baseline data.

## Conclusions

Our findings indicate that there is an association between DM and anemia among Korean adults, with a strong association between DM and anemia among men. In addition, the serum creatinine level was identified as a factor that has a significant influence on DM and anemia among women. However, considering that the survey did not particularly investigate anemia, the mechanism could not be identified. As anemia may influence DM-related complications such as kidney disease and other cardiovascular diseases, management and treatment of anemia in patients with DM are considered highly important.

## Supplementary Information

**Additional file 1.** Associations between DM and sex and interaction between DM and sex in anemia

## Data Availability

The datasets generated and analyzed during the current study are available in the KNHANES repository (http://knhanes.cdc.go.kr). All data from the KNHANES V, VI, and VII are coded and freely available.

## Abbreviations

BMI: Body mass index
CI: Confidence interval
DM: Diabetes mellitus
FBS: Fasting blood sugar
GFR: Glomerular filtration rate
Hb: Hemoglobin
HbA1c: Glycated hemoglobin
HDL: High-density lipoprotein
KNHANES: Korea National Health and Nutrition Examination Survey
OR: odds ratio
