# Supplementary material for: Association between diabetes mellitus and anemia among Korean adults according to sex: a cross-sectional analysis of data from the Korea National Health and Nutrition Examination Survey (2010–2016)
Source: BMC Endocr Disord. 2021 Oct 21;21:209. doi: 10.1186/s12902-021-00873-9 (PMC8529722; doi:10.1186/s12902-021-00873-9)
Supplement: Supplementary file 1 — Additional file 1. Associations between DM and sex and interaction between DM and sex in anemia [file 12902_2021_873_MOESM1_ESM.docx]

**Additional file 1.** Associations between DM and sex and interaction between DM and sex in anemia ^a^

|  | Model 1 | | |  | p-value | Model 2 | |  | p-value | Model 3 | |  | p-value |  |
| --- | --- | --- | --- | --- | --- | --- | --- | --- | --- | --- | --- | --- | --- | --- |
|  | OR ^b^ | | (95% CI) | |  | OR ^b^ | (95% CI) | |  | OR ^b^ | (95% CI) | |  |  |
| DM | 3.49 | 2.69-4.53 | | | <.0001 | 3.10 | 2.38-4.04 | | <.0001 | 2.97 | 2.27-3.89 | | <.0001 |  |
| Sex | 5.52 | 4.73-6.45 | | | <.0001 | 4.36 | 3.70-5.14 | | <.0001 | 5.66 | 4.61-6.95 | | <.0001 |  |
| DM*Sex | 0.35 | 0.25-0.49 | | | <.0001 | 0.37 | 0.27-0.52 | | <.0001 | 0.39 | 0.27-0.54 | | <.0001 |  |
| ^a^ Hb level was used as the indicator of anemia. Based on the criteria put forth by the World Health Organization, anemia was defined as an Hb level <13 g/dL in men and <12 g/dL in women.  ^b^ Odds ratios with adjustments using logistic regression models.  Model 1: unadjusted.  Model 2: adjusted for age, sex, household income, educational level, body mass index, smoking status, and alcohol consumption.  Model 3: adjusted for age, sex, household income, educational level, body mass index, smoking status, alcohol consumption, iron intake per day, serum creatinine level, high waist circumference, hypertension, hypertriglyceridemia, and low high-density lipoprotein cholesterol level.  **DM**, diabetes mellitus; **OR**, odds ratio; **CI**, confidence interval | | | | | | | | | | | | | | |
